# Dog-assisted interventions to support health and wellbeing: a national survey of current practice in England

**DOI:** 10.3389/fpubh.2025.1645811

**Published:** 2025-08-05

**Authors:** Emily Shoesmith, Sophie S. Hall, Evgenia Riga, Daniel S. Mills, Selina Gibsone, Dean McMillan, Qi Wu, Chris Clarke, Elena Ratschen

**Affiliations:** ^1^Department of Health Sciences, University of York, Heslington, United Kingdom; ^2^Nottingham Clinical Trials Unit, University of Nottingham, Nottingham, United Kingdom; ^3^Department of Life Sciences, University of Lincoln, Lincoln, United Kingdom; ^4^Dogs for Good, The Frances Hay Centre, Banbury, United Kingdom; ^5^Foss Park Hospital, Tees, Esk and Wear Valleys NHS Foundation Trust, York, United Kingdom

**Keywords:** dog-assisted intervention, England, national survey, dog training, dog welfare, barriers, facilitators

## Abstract

**Introduction:**

In England, dog-assisted interventions (DAIs) are increasingly used to support the health and wellbeing of individuals with mental and physical health conditions. Although research in this area is increasing, little is known about the national prevalence and characteristics of DAIs in practice. Advancing knowledge is important to inform development of research, policy and practice in the field.

**Methods:**

An online survey of DAI providers in England was conducted to collect data on DAI service provision, including target populations, session content and structure, implementation challenges, and best practices in dog selection, training, and welfare monitoring.

**Results:**

Of 72 invitations sent to DAI organizations and therapists, 31 participants completed the survey (response rate: 41.3%). DAIs were primarily used to support individuals with mental health and neurodevelopmental conditions (e.g., anxiety, depression, autism) across the National Health Service (80.6%), private healthcare (61.3%), and educational settings (41.9%). Respondents estimated delivering DAIs across 7,679 institutions. Interventions included structured therapeutic sessions (61.3%) and spontaneous activities (51.6%), mostly delivered individually (90.5%) rather than to groups. Over half reported delivering dog-assisted therapy, but 38.7% were unsure whether sessions had individualized goals. Session frequency and duration varied; most lasted 31–60 min (54.8%) and occurred weekly (45.2%). Key challenges included limited access to delivery spaces (35.5%), infection control concerns (32.3%), and difficulties “matching” dogs to service users (22.6%). Dog selection relied on temperament assessments (100%). While 54.8% of handlers received formal training, content varied. Some were trained in dog body language and risk assessment; others were not. Welfare monitoring primarily depended on handler observation (90.3%) and access to basic resources such as water.

**Discussion:**

DAIs are now implemented in thousands of health, care and educational settings across England, serving diverse clinical populations of all ages, especially those with mental health and neurodevelopmental conditions. Interventions range from structured, goal-oriented sessions (e.g., supporting mental, emotional, or physical health) to spontaneous interactions (e.g., community programmes, social events, recreational activities). Participant responses highlight substantial variation in DAI characteristics and delivery. These findings underscore the clear need for standardized good practice guidelines encompassing aspects related to outcome reporting, dog selection and welfare monitoring, and provider training.

## Introduction

In England and many other countries worldwide, demand, provision and research of animal-assisted interventions (AAIs) to improve human health and wellbeing has been rapidly growing (1–7). Although a variety of species (e.g., dogs, horses, small mammals, farm animals) are involved in AAI research and practice, dog-assisted interventions (DAIs) are currently the most commonly provided and researched type of AAI (8). DAIs can include dog-assisted therapy (DAT), which is goal-orientated, structured and delivered by certified therapists (7, 9, 10), whereas dog-assisted activities (DAAs) are typically based on spontaneous interaction and do not have documented, defined outcomes (7, 10).

Despite the expanding interest and research activity surrounding DAIs, there remains a limited understanding of the prevalence, nature, and characteristics of DAI provision within England. While health and safety guidelines for implementing DAIs have been developed across multiple sectors (e.g., healthcare, education, voluntary organizations) (11, 12), the overall provision of DAIs remain largely unregulated (13). Moreover, there is an absence of uniformly accepted standards, procedures, or policies to govern the delivery of these interventions, leading to considerable variability in practice (14, 15). While surveys reporting on real-life practices of AAIs have been conducted in the United States (14, 15), no comparable studies, except one focusing on AAIs in intensive care units (16), have been undertaken in England.

Understanding current practices and standards of DAI provision is critical not only for effective implementation and the safe and ethical delivery of these interventions, but also for enhancing the quality and transparency of research in this field. While a growing body of literature demonstrates the potential benefits of DAIs across diverse clinical populations and age groups (17–23), the extent to which these findings reflect real-world practice remains unclear. Little is known about how DAIs are implemented in a real-world context, including session content and structure, how dog-handler teams are selected and trained, and adherence to existing guidelines (7). This disconnect may suggest that a discrepancy exists between the controlled environments of research and the realities of service provision, highlighting the need for data on real-world practices.

Understanding more about the landscape of DAI provision in England will also facilitate international comparisons and the exchange of best practices, ensuring a more cohesive and informed approach to intervention development and evaluation. Therefore, the aim of the reported study was to undertake a survey of DAI service provision and practice in England.

## Methods

### Study design

A cross-sectional online survey was distributed to DAI providers in England.

### Recruitment and procedures

The survey was released in Qualtrics survey software (Qualtrics, Provo, UT, USA). Organizations specializing in the provision of DAIs in England (e.g., Dogs for Good, Pets as Therapy) were identified based on the researchers' multi-sector networks and extensive internet searches. These searches were conducted using Google with combinations of key terms such as “dog-assisted therapy,” “dog-assisted activity,” and “animal-assisted interventions” and restricted to results in England. We also manually reviewed directories and public listings of third-sector and healthcare organizations known to deliver or promote DAIs. Additionally, the British Association for Counseling and Psychotherapy Therapist Directory was searched to identify therapists with registered expertise in delivering DAIs. Personalized email invitations were sent to organizations and therapists, with up to two follow-up reminders. For organizations, only one participant was permitted to complete the survey on behalf of each organization to avoid duplication. Participants were asked to self-nominate or be nominated by their organization as the most appropriate person to complete the survey based on their familiarity with DAI delivery practices. To be eligible to participate, participants were required to be affiliated with an organization delivering DAIs in England, rather than those involved in the placement of assistance dogs.

Prospective participants were invited to follow a link to the survey where they were presented with a Participant Information Sheet (PIS) and consent form. Consent to participate was indicated by ticking an online check box. The PIS described a survey incentive being offered (opportunity to enter a prize draw with three participants randomly selected to win shopping vouchers). Screening questions required participants to confirm their involvement in DAI delivery and residence in England. All data were stored on the Qualtrics server of the University of York.

The survey opened for recruitment on September 2nd, 2024, and closed on January 13th, 2025. Approval for this research was granted by the Health Sciences Research Ethics Committee at the University of York, UK, on 29th September 2023 (Ref: HSRGC/2023/586/E).

### Measures

A bespoke survey was developed to enable collection of data most relevant to understanding the types and characteristics of DAI service provision in England. It was piloted by an expert group comprising a multi-disciplinary team of academics (*n* = 7), third sector animal welfare organizations (*n* = 3), and DAI providers (*n* = 3). Based on comprehensive feedback, a number of reinfements were made to the survey, including the addition of pre-determined response options, revisions to the definitions of dog-assisted therapy and activities to improve clarity, and adjustments to the survey logic to enhance flow and usability. The revised version was subsequently reviewed and piloted again to ensure that changes had been implemented as intended. Questionnaire items are summarized below; the full instrument is presented in Supplementary material 1.

#### Demographic data

Demographic information was gathered about participants' age (in ranges), gender (male/female/non-binary), their primary role/position within their organization (e.g., professional/paid animal handler, director, volunteer, occupational therapist, clinician, teacher, social worker), and number of years' experience of providing DAIs.

#### Organizational information

Information was gathered about the respondent's affiliated DAI organization. If the participant worked at more than one organization, they were asked to answer questions with their primary DAI-providing organization in mind. Information was collected on organization type (e.g., third sector/charity organization, local authority/council, NHS organization, private hospital, residential care and nursing home, hospice, school, nursery or pre-school); the number of paid and volunteer dog handlers registered at the organization, and not-for-profit status (yes/no).

#### DAI settings and target populations

Information was collected regarding the settings in which organizations currently delivered DAIs. Options included NHS mental health settings (e.g., adult mental health services, older people's mental health services, child and adolescent mental health services); NHS physical health settings (e.g., GP practices, outpatient clinics, inpatient wards, intensive care units); non-NHS health and care settings (e.g., hospices, care/nursing homes), and educational settings (e.g., nurseries, pre-schools, schools, universities). Participants were asked to provide the approximate number of settings or institutions in which their organization currently provided DAIs, and, if known, to provide this number.

Participants were also asked to identify the target populations to whom DAIs were delivered. Response options included adults and/or children and young people with physical health conditions; adults with mental health and neurodevelopmental conditions [e.g., anxiety, depression, schizophrenia, post-traumatic stress disorder, autism, eating disorders, attention-deficit/hyperactivity disorder (ADHD), intellectual disabilities]; children with mental health and neurodevelopmental conditions (e.g., anxiety, depression, emerging behavioral problems, autism, ADHD, eating disorders, trauma, attachment-related difficulties, intellectual disabilities); and adults with neurodegenerative conditions (e.g., dementia).

#### DAI types, frequency, duration, and session structure

Based on a brief list of definitions provided within the survey, participants were asked to identify what kind of DAIs their organization provided: dog-assisted therapy (i.e., goal-directed, documented, therapeutic intervention as part of a treatment plan, delivered by specialist-trained dog-handler teams), dog-assisted activities (i.e., activities focused on spontaneous interactions that can be delivered by volunteers and untrained dogs), both, or other. Dog-assisted therapy was defined as goal-directed interactions with dogs, led by trained professionals, and dog-assisted activities were defined as spontaneous interactions with dogs, often led by volunteers. If participants indicated their organization delivered dog-assisted therapy, they were asked to specify whether these sessions had clear individualized goals for the service user. We do acknowledge the recent suggestion of the new terminology [animal-assisted services; AASs (24)] within the survey (see Supplementary material 1). However, items were developed in collaboration with our expert group, who advised the new terminology may not yet be nationally recognized in the UK and so may confuse respondents. Therefore, we opted to use the original terminology in the context of this survey.

Participants were also asked to indicate whether DAIs are typically delivered on an individual basis or in group settings (e.g., with peers and/or family members). Additional information was collected regarding the frequency of DAI delivery (e.g., multiple times per week, once per week, more than once every 2 weeks but less than weekly, every 2 weeks, or varying by service user); the typical duration over which DAIs were provided (in weeks), and the usual length of individual sessions (e.g., 30 min or less, 31 min to 1 hour, more than 1 h but < 2, 2 h or more).

#### Dog-handler team training

##### Handler training

Participants were asked whether handlers within their organization received formal training (yes/no), and if so, to indicate the training received among nine pre-specified options, or to specify it themselves. The pre-specified training options included: “service user wellbeing/safety”; “dog wellbeing/safety”; “dog obedience/skill training”; “risk assessment and awareness”; “human-dog bond” (e.g., exercises to build mutual trust and responsiveness between the dog and handler, such as eye contact games or joint play activities); “dog body language”; “general mental health”; “condition/diagnosis-specific training,” and “safeguarding.”

##### Handler confidence

Participants were then asked to indicate their confidence level within their role, using a ten-point Likert scale (1 = not at all confident; 10 = very confident). An option was included for participants to leave an open-ended, free-text comment to share further information on their confidence level within their role.

##### Dog training

Participants were asked to indicate what training a dog receives prior to working, selecting from six pre-specified options, or to specify themselves. The pre-specified options included: “obedience training (e.g., leadwork, sit, down)”; “handling training (e.g., to be stroked/touched by strangers); greeting training (e.g., to greet people calmly)”; “human-dog bond”; “trick training,” or “socialization training in environments likely to work in.”

#### Dog characteristics, selection and welfare

##### Dog characteristics

Participants were asked to indicate what type of dogs are typically involved in the DAIs using Kennel Club groups (25), the youngest age a dog may start working, and the oldest age a dog will normally work until.

##### Dog selection

Participants were asked to specify how a dog is selected for work, selecting from the following options: “temperament/behavior assessment”; “pass obedience test [e.g., Kennel Club test (26)]”; “health status (pass veterinary assessment)”; “specific characteristics (e.g., size, color, breed, age),” or for the participant to specify their own reason.

Information was collected about whether the dogs needed to have any health checks passed prior to their involvement in DAIs (yes/no), and if so, to indicate the health checks among six pre-specified options, or to specify it themselves. The pre-specified health check options included: “annual vaccinations”; “regular worming programme”; “not currently receiving any veterinary-prescribed medication”; “no current health issues that have prohibited the issuing of veterinary sign off”; “regular grooming schedule,” and “not on a raw food diet.”

##### Dog welfare

Information was gathered regarding how many handlers, on average, the dogs work with; how frequently a dog is involved in the DAI (daily, a few days a week, weekly, less than once a week), and whether there is a maximum number of hours a dog can work per week (yes/no). Participants were also asked to indicate whether strategies are in place to ensure the dog's welfare is monitored, and if so, to indicate the strategies among five pre-specified options, or to specify it themselves. The pre-specified options included: “handler observation”; “veterinary observation”; “using checklists or other written materials”; “access to water,” and “access to a place the dog can choose to go by themselves.”

Participants were also asked whether a formal process was in place for reporting incidents of inappropriate dog behavior or injuries to either human participants or the dog (yes/no).

##### Barriers and facilitators to DAI provision

Participants were asked whether they experienced barriers when delivering DAIs, and if so, to indicate the reason among seven pre-specified options, or to specify it themselves. The pre-specified barriers included: “controlling potential risk factors and infection prevention control”; “access to appropriate space for delivery”; “challenges related to monitoring dog welfare”; “challenges related to matching dogs to service users”; “working with other professions”; “lack of appropriate skills/training for dog,” and “challenges related to access to appropriate training for handler.” A free-text, open-ended box was provided for participants to identify the most important facilitators for successful implementation.

### Data analysis

Data were analyzed in SPSS v29.0 (IBM SPSS Inc., Chicago, USA) (27) and descriptive summary statistics are presented for demographic variables and data relating to the nature and characteristics of DAI provision, settings and target populations, session structure, dog-handler team training, dog characteristics, selection and welfare, and implementation barriers and facilitators. Categorical variables were analyzed using frequencies and percentages, while continuous variables were analyzed using means, standard deviations, and ranges. Free-text response content was analyzed descriptively to identify common themes and frequently mentioned issues across participants.

## Results

Of the 72 invitations sent to DAI organizations (*n* = 44) and therapists (*n* = 28), 40 individuals accessed the survey link. Two respondents were excluded due to ineligibility, as they were based outside of England, resulting in 38 eligible participants who consented to participate in the survey. Of these, six were excluded from analysis for not completing the survey beyond the initial screening questions. Additionally, one response was identified as a duplicate, as two members of the same organization completed the survey. Therefore, the final sample comprised 31 participants, all of whom completed the questionnaire in full, with no missing data (response rate = 43.1%). A summary of participant and organizational characteristics is presented in Table 1.

**Table 1:** Participant and organizational characteristics.

**Participant characteristics**	***N* (%)**
**Gender**	
Female	29 (90.3)
Male	2 (6.5)
Prefer not to say	1 (3.2)
**Age (years)**	
18–25	1 (3.2)
26–34	2 (6.5)
35–44	5 (16.1)
45–55	12 (38.7)
56–65	9 (29.0)
Over 65	2 (6.5)
**Primary role**	
Clinician (e.g., consultant, psychologist, mental health nurse)	15 (48.4)
Professional/paid animal handler	5 (16.1)
CEO/Director	4 (12.9)
Volunteer animal handler	4 (12.9)
Occupational therapist	3 (9.7)
**Years of experience**	
< 1 year	2 (6.5)
1–3 years	7 (22.6)
4–7 years	9 (29.0)
≥8 years	13 (41.9)
**Organizational characteristics**	***N*** **(%)**
**Region**	
South East England	9 (29.0)
North West England	7 (22.6)
South West England	6 (19.4)
West Midlands	4 (12.9)
North East England	3 (9.7)
East Midlands	1 (3.2)
South Central	1 (3.2)
**Organization type**	
Counseling/psychotherapy practice	13 (41.9)
Third sector/charity organization	12 (38.7)
School	4 (12.9)
NHS organization	2 (6.5)
**Not-for-profit**	
No	18 (58.1)
Yes	12 (38.7)
Not sure	1 (3.2)
1–2	19 (61.3)
3–5	2 (6.5)
6–10	1 (3.2)
11–20	1 (3.2)
Not sure	8 (25.8)
**Volunteer dog handlers**	
1–2	12 (38.7)
3–5	2 (6.5)
6–10	1 (3.2)
11–20	1 (3.2)
21+	4 (12.9)
Not sure	11 (35.5)

### DAI settings and target populations

Most participants reported that their organizations delivered DAIs in non-NHS health and care settings (*n* = 25; 80.6%), followed by educational settings (*n* = 13; 41.9%), NHS mental health services (*n* = 10; 32.3%), and NHS physical health services (*n* = 9; 29.0%). NHS settings refer to services provided within the publicly funded National Health Service, whereas non-NHS settings operate outside this system, often under private or third-sector provision.

With non-NHS settings, DAIs were most commonly reported to be provided in mental health settings (*n* = 14), care or nursing homes (*n* = 9), physical healthcare settings (*n* = 8), assisted living facilities (*n* = 6), and hospices (*n* = 5). Educational settings included schools (*n* = 12), universities (*n* = 7), pre-schools (*n* = 5), and nurseries (*n* = 4). Among NHS mental health settings, DAIs were delivered in adult and older adult mental health services (each *n* = 7), child and adolescent mental health services (*n* = 5), and forensic services (i.e., secure wards; *n* = 5). In NHS physical healthcare settings, delivery occurred primarily in inpatient wards (*n* = 9), followed by intensive care units (*n* = 6), outpatient clinics (*n* = 5), and general practice (GP) clinics (*n* = 2).

Twenty-one participants (67.7%) reported they were aware of the approximate number of institutions (e.g., hospitals, health services, community centers) where their organization provided DAIs. Estimates ranged from 1 to 6,000 institutions, with a cumulative total of 7,679 institutions across all participants. This wide range reflects the diversity in organizational size and reach among respondents, including both smaller local providers and some of the largest DAI organizations operating nationally within the UK.

Populations served varied substantially across organizations, as DAIs were delivered to adults with a mental health or neurodevelopmental condition (*n* = 21; 67.7%), a physical health condition (*n* = 13; 41.9%), and/or a neurodegenerative condition (*n* = 13; 41.9%). Organizations also delivered DAIs to children and young people with a mental health or neurodevelopmental condition (*n* = 19; 61.3%) or a physical health condition (*n* = 9; 29.0%). Table 2 provides an overview of service user diagnoses.

**Table 2:** Overview of service user diagnoses.

**Diagnosis**	***N* (%)**
**Adults with mental health/neurodevelopmental conditions**	
**(*****n*** = **21)**	
Anxiety and/or depression	21 (100.0)
Autism spectrum condition	19 (90.5)
Post-traumatic stress disorder	18 (85.7)
Attention deficit and hyperactivity disorder	17 (80.9)
Eating disorder	10 (47.6)
Schizophrenia and/or other severe mental illness	10 (47.6)
Intellectual disability	10 (47.6)
Other	5 (23.8)
**Adults with neurodegenerative conditions (*****n*** = **13)**	
Dementia	13 (100.0)
**Adults with physical health conditions (*****n*** = **13)**	
Various (e.g., epilepsy, cancer, diabetes)	7 (53.8)
Confidential/not known	2 (15.4)
Acquired brain injuries	2 (15.4)
Mobility related (e.g., cerebral palsy, multiple sclerosis)	2 (15.4)
**Children/young people with mental health/**	
**neurodevelopmental conditions (*****n*** = **19)**	
Anxiety and/or depression	18 (94.7)
Autism spectrum condition	18 (94.7)
Attention deficit and hyperactivity disorder	18 (94.7)
Trauma	15 (78.9)
Problems relating to attachment	14 (73.7)
Emerging behavioral problems	12 (63.2)
Intellectual disability	12 (63.2)
Eating disorder	10 (52.6)
Other	3 (15.8)
**Children/young people with physical health conditions (*****n*** = **9)**	
Various (e.g., epilepsy, cancer, diabetes)	7 (77.8%)
Acquired brain injuries	1 (11.1%)
Confidential/not known	1 (11.1%)

### DAI types, frequency, duration and session structure

The majority of participants reported their organization delivered dog-assisted therapy (i.e., goal-oriented interactions with dogs, led by trained animal-handler professionals and/or certified therapists; *n* = 19; 61.3%), whereas just over half reported delivering dog-assisted activities (i.e., spontaneous interactions with dogs, often led by volunteers; *n* = 16; 51.6%). When asked how often the DAI session had clear goals for the user, seven participants (22.6%) indicated “always,” six participants (19.4%) reported “most of the time,” six participants (19.4%) reported “sometimes,” and 12 (38.7%) reported they were unsure/did not know.

Regardless of intervention type or population served, DAIs were most frequently delivered on an individual basis (*n* = 28; 90.3%), followed by group-based delivery with peers (*n* = 16; 61.6%), and group-based delivery with relatives (*n* = 6; 19.4%). While 18 participants (58.1%) reported that the duration of DAIs “varied considerably” depending on the population group and their specific needs, seven participants (22.6%) typically delivered DAIs for a duration of 6–10 weeks. Other reported durations included 1–5 weeks (*n* = 3; 9.7%) and 11–15 weeks (*n* = 3; 9.7%). Likewise, nine participants (29.0%) indicated that the frequency of DAI sessions per service user “varied considerably.” However, for those reporting a consistent frequency, the most common was once a week (*n* = 14; 45.2%), followed by multiple times per week (*n* = 7; 22.6%). Only one participant (3.2%) stated that their organization delivered sessions once every 2 weeks. The average length of a DAI session was frequently reported as 31 min – 1 h (*n* = 17, 54.8%). However, nine participants (29.0%) reported sessions could last anywhere from >1 to < 2 h, and five participants (16.1%) reported their sessions were 30 min or less.

### Dog-handler team training

Just over half of the participants (*n* = 17; 54.8%) reported the dog handlers received formal training. Among those, the most frequently reported training included dog wellbeing and safety (94.1%), general risk assessment and awareness (94.1%), service user wellbeing and safety (88.2%), and recognizing dog body language (88.2%). A full overview of the training reported is presented in Table 3.

**Table 3:** Dog handler training.

**Dog handler training**	***N* (%)**
Dog wellbeing/safety	16 (94.1)
General risk assessment and awareness	16 (94.1)
Service user wellbeing/safety	15 (88.2)
Dog body language	15 (88.2)
Safeguarding	15 (88.2)
General mental health	14 (82.4)
Dog obedience/skill training	13 (76.5)
Human-dog bond	13 (76.5)
Condition/diagnosis-specific training	11 (64.7)

When participants were asked to rate their confidence within their role on a 10-point Likert scale (1 = not at all confident; 10 = very confident), the mean score was 8.7 (range = 5–10, standard deviation = 1.26).

In addition to handler training, participants also reported a range of training activities undertaken by dogs prior to their involvement in DAIs. The most frequently reported training activities included basic obedience (e.g., leadwork, sit, down; 80.6%), handling training (77.4%), and greeting training (74.2%). Socialization in environments similar to those in which the dogs would work (74.2%) and training to support the development of the human-dog bond (67.7%) were also commonly reported. A full summary of reported training types is provided in Table 4.

**Table 4:** Training dog receives before starting DAI sessions.

**Dog training**	***N* (%)**
Obedience training (e.g., leadwork, sit, down, etc.)	25 (80.6)
Handling training (e.g., to be stroked/touched by strangers)	24 (77.4)
Greeting training (e.g., to greet people calmly)	23 (74.2)
Socialization training in environments likely to work in	23 (74.2)
Training related to human-dog bond development	21 (67.7)
Trick training	11 (35.5)

### Dog characteristics, selection, and welfare

Participants reported that dogs involved in DAI sessions were most commonly from the following UK Kennel Club classifications: Gundog group (e.g., Labradors, Golden Retrievers, Spaniels; *n* = 15; 48.4%) and crossbreeds (*n* = 15; 48.4%). Other represented classifications included the Working group dogs (e.g., Boxers, Dobermans, Rottweilers; *n* = 9; 29.0%), and Toy group dogs (e.g., Cavalier King Charles Spaniels, Chihuahuas; *n* = 8; 25.8%). Those less commonly reported included Terrier group dogs (e.g., Border Terriers; *n* = 7; 22.6%), Hound group dogs (e.g., Beagles, Greyhounds; *n* = 6, 19.4%), Pastoral group dogs (e.g., Border Collies, German Shepherds; *n* = 6; 19.4%), and Utility group dogs (e.g., Bulldogs, Dalmatians; *n* = 5; 16.1%).

Regardless of dog type, the youngest age at which the dog would start DAI work differed across organizations. Thirteen participants (41.9%) reported the youngest age a dog would commence work was 7–12 months, whereas others reported >2 years (*n* = 5; 16.1%); ≤ 6 months (*n* = 5; 16.1%); 13–18 months (*n* = 4; 12.9%), or 19–24 months (*n* = 4; 12.9%). Eight participants (25.8%) reported that there was no maximum age limit for dogs to continue working, while 10 participants (32.3%) indicated that the maximum age for a working dog would be 10 years. Five participants (16.1%) stated that the maximum age would be < 10 years, and four participants (12.9%) reported it would be over 10 years. Additionally, four participants (12.9%) were unsure whether there was a maximum age limit for the dogs involved.

When selecting dogs for DAIs, all participants (100%) reported temperament/behavior assessment as a selection criterion. Additional selection criteria were identified, including health status (passing veterinary assessment; *n* = 14; 45.2%); passing obedience test (e.g., Kennel Club test; *n* = 10; 32.3%), and selecting dogs based on specific characteristics (e.g., size, color, breed, age; *n* = 6; 19.4%).

In addition to these selection criteria, 26 participants (83.9%) reported the type of health checks in place for dogs prior to or during their involvement (Table 5). In addition to the pre-determined options, one participant reported in a free-text box that “*the dog must have been neutered/spayed*,” and another indicated “*there must be proof of immunity as per the World Small Animal Veterinary Association* (28)*.”*

**Table 5:** Type of dog health checks.

**Dog health checks**	***N* (%)**
Regular worming programme followed	26 (100.0)
Annual vaccinations	24 (92.3)
Regular grooming schedule followed	21 (80.8)
No current health issues (that have prohibited the issuing of veterinary sign off/approval for involvement in DAIs)	19 (73.1)
Not on a raw food diet	15 (57.7)
Not receiving any veterinary-prescribed medication	12 (46.2)

To monitor the wellbeing of dogs involved in DAIs, the most commonly reported strategies included handler observation (90.3%), ensuring access to water (87.1%), and providing a space the dog could choose to retreat independently (74.2%). Fewer organizations reported using tools such as checklists or written materials (32.2%) or veterinary or specialist observation (16.1%). The full set of reported wellbeing monitoring strategies are summarized in Table 6. Where participants indicated veterinary or other specialist observations were conducted (*n* = 5), the frequency ranged from every 3 months (*n* = 2) to every 6 months (*n* = 2), to twice a year (*n* = 1).

**Table 6:** Wellbeing monitoring strategies.

**Wellbeing monitoring strategies**	***N* (%)**
Handler observation	28 (90.3)
Access to water	27 (87.1)
Access to a place the dog can choose to go by themselves	23 (74.2)
Checklists/written materials	10 (32.2)
Veterinary or other specialist observation	5 (16.1)

Lastly, the majority of participants (*n* = 25; 80.7%) reported their organization had a process to report undesirable dog behavior/participant injury, whereas three participants (9.7%) indicated no processes were in place, and three participants (9.7%) were unsure. Participants who reported the presence of a formalized process in free-text responses frequently described procedures including dog assessments, incident reporting through accident books, session recording forms, and the reporting of incidents or concerns to a designated point of contact, in accordance with established safeguarding protocols.

### Barriers and facilitators to DAI provision

While six participants (19.4%) reported no perceived barriers to DAI implementation, others reported several challenges. The most frequently cited barriers included limited access to appropriate delivery spaces (35.5%), infection prevention and control concerns (32.3%), and challenges in appropriately matching dogs to service users (22.6%). Additional issues included difficulties in monitoring dog welfare and interprofessional collaboration. A full overview of the reported barriers are presented in Table 7.

**Table 7:** Perceived barriers to DAI implementation.

**Perceived barriers**	***N* (%)**
Access to appropriate space for delivery	11 (35.5)
Controlling potential risk factors and infection prevention control	10 (32.3)
Challenges related to matching dog(s) to service users	7 (22.6)
Challenges related to monitoring dog welfare (e.g., stress, fatigue)	6 (19.4)
Working with other professions	6 (19.4)
Challenges related to access to appropriate training for handler	4 (12.9)

Facilitators reported by free text included: a highly trained dog and handler adhering to animal welfare practices, an appropriate matching process to ensure a suitable dog-service user “fit,” allowing service users sufficient time to develop a relationship with the dog, and regular evaluation throughout implementation.

## Discussion

To our knowledge, this survey provides the first attempt to report on the types and characteristics of current DAI practice in England. Results indicate that DAIs have been implemented in thousands of health, care and educational settings, including NHS and non-NHS health and care settings, as well as schools, and are delivered across a range of mental and physical health conditions for both children and adults. Our findings reveal substantial variation in the delivery of DAIs, particularly in the service settings, training of handlers and dogs, and approaches to monitoring both human and animal welfare. These insights contribute to the ongoing discourse on best practice and the need for standardized guidelines in the field.

The majority of participants reported implementing dog-assisted therapy (goal-orientated, structured involving clinical professionals and trained dog handlers) just over half the sample also reported using dog-assisted activities (spontaneous interaction, often no defined outcomes). This suggests that while therapeutic interventions involving dogs are prevalent, there is also a considerable focus on non-therapeutic activities aimed at enhancing general wellbeing. However, it could also be a reflection of the lack of uniformity in defining and categorizing AAIs, as highlighted in previous research (24). In response to criticisms in the field regarding inconsistent taxonomy and definitions (29, 30), there have been recent efforts to propose uniform terminology that accurately reflect the key features of different AAI approaches within this field to ensure the appropriate preparation, training, and support of all parties involved and improve clarity for those involved in the delivery and receipt of AAIs (24). This approach proposes that animal-assisted services (AASs) should be used as an over-arching term, which is further categorized into three main areas: treatment, education, and support programs (24).

We found that most participating providers served individuals with mental health and neurodevelopmental conditions, particularly autism, ADHD, anxiety/depression, and PTSD. This aligns with existing evidence that suggests DAIs may be particularly beneficial for these populations (3, 17, 31, 32). It was not possible to conduct a meaningful comparison of survey responses across population groups due to the small sample size. Additionally, our expert group that details of the intervention are likely to be determined based on individual needs rather than the needs associated with a specific condition. Across target population groups, DAIs were primarily delivered on an individual basis, with fewer organizations providing group-based interventions. This suggests that a personalized approach is favored, potentially to better address individual needs and facilitate stronger human-animal bonds. Notably, session goals varied in consistency, with only a quarter of respondents indicating that sessions “always” had clear goals, and a substantial proportion (38.7%) reporting they did not know or were unsure. This inconsistency may reflect differences in organizational structures, training levels, or philosophical approaches to DAI implementation.

The reported frequency and duration of DAIs also varied considerably. While 45.2% of organizations provided sessions once per week, others offered multiple sessions per week or had variable schedules based on user needs. Similarly, while most sessions lasted between 31 min and 1 h (54.8%), others extended beyond 1 h or were shorter than 30 min. The findings on frequency and duration variability are congruent with a recent survey conducted in intensive care unit settings in the UK (16), indicating this may be common practice across a range of settings. These variations highlight the importance of flexibility in DAI programming, allowing for adaptations based on service user requirements and organizational capacity. However, they also highlight the importance of clear definitions and frameworks to ensure consistency in practice and research, depending upon the needs of the service users, service capacity and the needs, behavior and welfare of the dog.

Comparable to previous research in the field, our findings identified Gundogs (e.g., Labradors, Golden Retrievers, and Spaniels) as the most commonly involved dog type followed by crossbreeds (33). Our findings also highlight the methods used for dog selection (e.g., temperament/behavior assessment, health status, obedience tests), which is an important observation given recent research indicates selection methods are seldom specified (33). This considerable variability in selection protocols reflects the absence of standardized criteria or validated tests for identifying dogs or dog-related characteristics which make them most suitable for specific types of DAIs, and aligns with variability reported in multiple studies (34–37).

Another key issue highlighted by this study is the variability in handler and dog training. Only 54.8% of handlers received formal training. There was a lack of consistency in the breadth of essential training provided, and notable gaps in important areas such as risk assessment and understanding of dog body language. This lack of consistent and comprehensive training raises concerns about the safe and ethical delivery of DAI implementation. Given the increasing recognition of the importance of handler competency in ensuring safe and effective DAI delivery (15), future efforts should focus on establishing educational training standards in terms of both content and quality assured qualifications. Dog welfare was also a critical consideration in DAI implementation, with organizations claiming to employ a range of wellbeing strategies, including handler observation, access to water, and designated rest areas. However, only a small proportion of providers reported formal veterinary assessments or structured welfare checklists. This variability raises concerns about the potential for undetected distress or fatigue in working dogs, which has been noted as a limitation in previous studies (38). The development of standardized welfare assessment tools could enhance the consistency of monitoring practices across organizations. Several challenges to DAI implementation were identified, and these are important to consider when designing a research study or when considering implementing DAIs in practice. Around a third of participants (35.5%) cited limited access to appropriate space as a barrier, while concerns regarding infection control and the complexities of matching dogs to service users were also noted. These findings align with existing literature emphasizing issues relating to welfare and infection control in AAIs (15, 16). Furthermore, ensuring dog welfare and securing appropriate training for handlers were identified as additional challenges, highlighting the need for robust training protocols and ethical oversight. In contrast, key facilitators of successful DAI implementation included the use of highly trained dogs and handlers, adherence to strict animal welfare practices, and a well-structured matching process between dogs and service users; although it should be noted that the scientific quality of the latter process remains unknown. Additionally, allowing sufficient time for relationship-building between the dog and service user, as well as regular evaluation throughout implementation, were identified as beneficial strategies. These findings reinforce the need for a comprehensive framework grounded in robust scientific evidence that integrates best practices in DAI delivery and focused on ensuring both service user benefit and animal welfare.

### Implications

We established that DAIs are now implemented in many thousands of health, care and educational settings across the UK. There is clear potential for the further development and refinement of DAIs. The variability in session structure, duration, and goal-setting suggests a need for standardized guidelines to enhance consistency, ethical provision, safety, and efficacy, while allowing for individualized adaptations. Additionally, addressing reported key barriers, such as access to appropriate operational spaces, could facilitate wider implementation of DAIs. Future research should explore the long-term outcomes of DAIs for service users, as well as the impact of intervention structure and frequency on therapeutic effectiveness. Moreover, further investigation into best practices for dog assessment, welfare monitoring and handler training would contribute to the robustness, ethical and sustainable development of DAIs.

### Limitations

This study represents one of the first surveys focused on DAI provision in England, offering valuable insights into current practices and standards. However, several limitations should be acknowledged. Firstly, the study relied on self-reported data, which will be subject to bias or inaccuracies. Additionally, only one individual per organization was invited to complete the survey, which may have introduced variability in response quality depending on the respondent's role (e.g., director vs. volunteer). Secondly, the small sample size (*n* = 31) and relatively low response rate (43.1%) may not fully capture the full spectrum of DAI practices across England. Indeed, it is likely that DAIs are currently being provided in many thousands more settings than we captured, and with even greater variability in the reported aspects. Future research should aim to expand the sample and incorporate qualitative methodologies to gain a deeper understanding of implementation challenges. Furthermore, despite free-text boxes available in the survey, there was an absence of in-depth free-text data, which would have allowed for a more comprehensive exploration of participants' responses. However, this decision was made intentionally to avoid the survey from becoming overly time-consuming and burdensome, particularly given the substantial number of specific items included. It should also be recognized that this survey was limited to those practicing DAIs in England. The results reported here may support future comparisons across countries, and across varying socioeconomic demographics.

## Conclusion

This study demonstrates an encouraging breadth and versatility of DAIs across health and care settings in England, reflecting their integration into services that support individuals of all ages and clinical backgrounds and a clear indication of high demand and acceptability. From structured therapeutic programmes to informal community-based activities, DAIs appear to be valued for their potential to enhance mental, emotional, and physical wellbeing. At the same time, our findings underscore the pressing need for more standardized and validated procedures, particularly in areas such as practitioner training and animal welfare monitoring, to ensure the safe, ethical, and effective delivery of these interventions. While previous research points to the promise of DAIs, especially for individuals with mental health and neurodevelopmental conditions, inconsistency in practice and reporting continues to present barriers to achieving reliable outcomes and scaling services. Moving forward, greater collaboration between researchers, practitioners, and policymakers will be critical to address these challenges and establish a cohesive framework for best practice. By doing so, we can ensure that the full potential of DAIs is realized, delivering meaningful and lasting impact for service users and the wider field.

## Data Availability

The raw data supporting the conclusions of this article will be made available by the authors, without undue reservation.
