# Supplementary material for: Dog-assisted interventions to support health and wellbeing: a national survey of current practice in England
Source: Front Public Health. 2025 Aug 5;13:1645811. doi: 10.3389/fpubh.2025.1645811 (PMC12361161; doi:10.3389/fpubh.2025.1645811)
Supplement: Supplementary file 1 [file Table_1.docx]

**Supplementary Material 1:** Survey Items

| **Provision of dog-assisted interventions (DAIs) in the UK** | | | |
| --- | --- | --- | --- |
| **SCREENING QUESTIONS** | | | |
| **The first questions relate to the organisation you are working for or are affiliated/aligned with (‘your organisation’ in short from this point onwards).** | | | |
| **SC.01** | **Where is your organisation based?** | England |  |
|  |  | - South East England |  |
|  |  | - North East England |  |
|  |  | - East Midlands |  |
|  |  | - West Midlands |  |
|  |  | - South Central |  |
|  |  | - East of England |  |
|  |  | - North West England |  |
|  |  | - South West England |  |
|  |  | Scotland |  |
|  |  | Wales |  |
|  |  | Northern Ireland |  |
|  |  | Other country | **__END** |
| **SC.02** | **Does your organisation provide dog-assisted interventions (DAIs)?**  *In the context of this study, DAIs are defined as interventions that are provided with a therapeutic purpose. They include dog-assisted therapy (goal-directed interactions with dogs, led by trained professionals) or dog-assisted activities (spontaneous interactions with dogs, often led by volunteers).* | Yes |  |
|  |  | No | **__END** |
| **About You and Your Organisation** | | | |
| **1** | **Please select your age category.** | 18-25 years |  |
|  |  | 26-34 years |  |
|  |  | 35-44 years |  |
|  |  | 45-55 years |  |
|  |  | 56-65 years |  |
|  |  | Over 65 years |  |
|  |  | Prefer not to say |  |
| **2** | **Please select which gender you identify as.** | Male |  |
|  |  | Female |  |
|  |  | Non-binary |  |
|  |  | Prefer not to say |  |
| **3** | **Please state the name of the** **organisation that you provide DAIs for. If you provide DAIs for more than one organisation, please answer these questions with the primary organisation in mind.** | Free text |  |
| **4** | **What is your current role or position within your organisation?** | Professional/paid animal handler |  |
|  |  | CEO/Director |  |
|  |  | Volunteer animal handler |  |
|  |  | Volunteer other |  |
|  |  | Occupational therapist |  |
|  |  | Clinician (e.g., consultant, psychologist, nurse practitioner) |  |
|  |  | Teacher |  |
|  |  | Social worker |  |
|  |  | SENCO |  |
|  |  | Other (please specify) |  |
| **5** | **How many years of experience of providing DAI do you have?** | <1 year  1-3 years  4-7 years  8 years or more |  |
| **6** | **Is your organisation a not-for-profit organisation?** | Yes |  |
|  |  | No |  |
|  |  | Not sure |  |
| **7** | **Approximately how many canine handlers are registered in your organisation?** | 1 – 2 |  |
|  |  | 3 – 5 |  |
|  |  | 6 – 10 |  |
|  |  | 11 – 20 |  |
|  |  | 21 or more |  |
|  |  | Not sure |  |
| **8** | **Which option best describes your organisation?** | Third sector/charity organisation |  |
|  |  | Local authority/Council |  |
|  |  | NHS organisation | Go to 11 |
|  |  | Private hospital | Go to 11 |
|  |  | Residential care and nursing home | Go to 11 |
|  |  | Hospice | Go to 11 |
|  |  | School | Go to 11 |
|  |  | Nursery or pre-school | Go to 11 |
|  |  | Other: please specify | Go to 11 |
| **9** | **Please tell us in what type of settings your organisation currently provides DAIs? Please select all that apply.** | 1. **NHS - Mental Health** |  |
|  |  | - Adult mental health services (AMHS) |  |
|  |  | - Older people’s mental health services (including dementia) |  |
|  |  | - Child and Adolescent Mental Health Services (CAMHS) |  |
|  |  | - Forensic services (i.e., secure wards) |  |
|  |  | - Recovery homes |  |
|  |  | - Other (please specify) |  |
|  |  | 1. **NHS - Physical Health** |  |
|  |  | - GP Practices |  |
|  |  | - Outpatient clinics |  |
|  |  | - Inpatient wards |  |
|  |  | - Intensive care units |  |
|  |  | - Other (please specify) |  |
|  |  | 1. **Non-NHS Health and Care** |  |
|  |  | Mental health |  |
|  |  | Physical health |  |
|  |  | Hospices |  |
|  |  | Care/nursing homes |  |
|  |  | Assisted living facilities |  |
|  |  | 1. **Education** |  |
|  |  | Nurseries |  |
|  |  | Pre-schools |  |
|  |  | Schools |  |
|  |  | Universities |  |
|  |  | Other (please specify) |  |
| **10a** | **10a. Do you know approximately how many institutions (e.g., health and care, education) your organisation currently provide DAIs in?** | a. Yes b. No/Not sure |  |
| **10b** | **[If yes] What is the approximate number of institutions your organisation currently provides DAIs in?** | [Text box allowing numbers only] |  |
| **11** | **Are any of the DAIs your organisation provides part of an Integrated Care Board (ICB) commissioning scheme or part of social prescribing (e.g., through GP referral)?** | Yes, we are funded by an ICB |  |
|  |  | Yes, we provide interventions as part of social prescribing |  |
|  |  | Yes, we are funded by an ICB and also provide DAIs as part of social prescribing |  |
|  |  | Neither |  |
|  |  | Not sure |  |
| **12** | **What kind of DAIs does your organisation provide? Please select all that apply or ‘not sure’ if you don’t know the answer to this question.** We acknowledge that a new uniform terminology for 'Animal-Assisted Services' (Binder et al., 2024) has recently been suggested and appreciate its importance, but due to its novelty we are using the traditional terminology in the context of this survey. | Dog-assisted therapy (i.e., goal-directed, documented therapeutic intervention as part of a treatment plan, delivered by specialist-trained dog-handler teams) | _**If selected, go to Q12b** |
|  |  | Dog-assisted activities (i.e., activities focused on spontaneous interactions that can be delivered by volunteers and untrained dogs, i.e., hospital or care home visits) |  |
|  |  | Other (please specify) |  |
|  |  | Not sure |  |
| **12b** | **Does each DAI session provided by you/your organisation have clear individualised goals for the service user?** | Always |  |
|  |  | Most of the time |  |
|  |  | Sometimes |  |
|  |  | Rarely |  |
|  |  | No |  |
|  |  | Not sure |  |
| **13** | **Are the DAIs you provide usually delivered to…: Please select all that apply** | Groups (peers) |  |
|  |  | Groups (families) |  |
|  |  | Individuals |  |
| **14** | **Typically, how often does a service user take part in a DAI delivered by your organisation?** | Multiple times per week |  |
|  |  | Once a week |  |
|  |  | More frequently than every two weeks but less than once a week |  |
|  |  | Every two weeks |  |
|  |  | Varies considerably per service user |  |
| **15** | **For how many weeks is a service user typically involved with a DAI?** | Give number of weeks – Drop down list, starting at 1 – 50 (and option to say it varies considerably). |  |
| **16** | **Who do you most frequently deliver DAIs to? Please select all that apply** | **Adults with physical health conditions** (please specify) |  |
|  |  | **Children and young people with physical health conditions** (please specify) |  |
|  |  | **Adults with mental health and neurodevelopmental conditions** |  |
|  |  | - Anxiety and/or depression |  |
|  |  | - Schizophrenia and/or other Severe Mental Illness (SMI) |  |
|  |  | - Post-Traumatic Stress Disorder (PTSD) |  |
|  |  | - Autism Spectrum Disorder (ASD) |  |
|  |  | - Eating disorders |  |
|  |  | - Attention Deficit and Hyperactivity Disorder (ADHD) |  |
|  |  | - Intellectual disabilities |  |
|  |  | - Other (please specify) |  |
|  |  | **Adults with neurodegenerative conditions** |  |
|  |  | Dementia |  |
|  |  | Other (please specify) |  |
|  |  | **Children and young people with mental health and neurodevelopmental conditions** |  |
|  |  | - Anxiety and/or depression |  |
|  |  | - Emerging behavioural problems |  |
|  |  | - Autism Spectrum Disorder (ASD) |  |
|  |  | - Attention Deficit and Hyperactivity Disorder (ADHD) |  |
|  |  | - Eating disorders |  |
|  |  | - Trauma |  |
|  |  | - Problems relating to attachment |  |
|  |  | - Intellectual disabilities |  |
|  |  | - Other (please specify) |  |
| **17** | **Please select the top 3 protected characteristics groups who you feel may be less likely to engage in DAIs:** | **Age (please state):** [Age categories as above] |  |
|  |  | **Gender (please state)**  Female  Male  Non-Binary/Third gender |  |
|  |  | **Disability (please state)**  Physical  Mental  Both  Other (Please specify if known) |  |
|  |  | **Marriage/civil partnership (please state)**  Single  In a civil partnership  Married  Separated/Divorced  Widowed  Other (Please specify if known) |  |
|  |  | **Pregnancy/maternity (please state)**  Pregnant  Post-Partum  Other (Please specify if known) |  |
|  |  | **Ethnicity (please state)**  White British, Irish, Other  Black, Black British  Asian, Asian British  Mixed  Other (Please specify if known) |  |
|  |  | **Religion/belief (please state)**  Christian  Muslim  Hindu  Sikh  Jewish Buddhist Agnostic/Atheist  Other (Please specify if known) |  |
|  |  | **Gender reassignment** (please state) |  |
|  |  | **Sex (please state)**  Female  Male  Intersex |  |
|  |  | **Sexual orientation (please state)**  Heterosexual  Bisexual Gay/Lesbian  Other (Please specify if known) |  |
|  |  | **Please provide any other information that you wish to share** |  |
|  |  | I am not sure |  |
| **18** | **On a scale of 1-10, how confident do you feel in your role within a DAI?** | Scale of 1-10  **If you would like to share any more information on your confidence level within your role, please use the text box below:**  **[**Include optional free-text box for participants to add further info should they wish] |  |
| **19** | **When delivering DAIs, which of these barriers do you commonly experience? Please select all that apply.** | Controlling potential risk factors and infection prevention control |  |
|  |  | Access to appropriate space for delivery |  |
|  |  | Challenges related to monitoring dog welfare (e.g., monitoring stress, fatigue) |  |
|  |  | Challenges related to matching dog(s) to service users |  |
|  |  | Working with other professions |  |
|  |  | Lack of appropriate skills/training for dog |  |
|  |  | Challenges related to access to appropriate training for handler |  |
|  |  | No barriers experienced |  |
|  |  | Other (please specify) |  |
| **20** | **What do you feel are the most important facilitators for a successful DAI?** | Free text |  |
| **Health & Safety** | | | |
|  | **The following questions are related to health and safety within your organisation.** | |  |
| **21** | **Do handlers have to pass a formal evaluation before delivering DAI in your organisation?** | Yes |  |
|  |  | No |  |
|  |  | Not sure |  |
| **22** | **Do handlers receive formal training? Yes/No**  **[If yes] What kind of training do handlers receive? Please select all that apply.** | Service user wellbeing/safety |  |
|  |  | Dog wellbeing/safety |  |
|  |  | Dog obedience/skill training |  |
|  |  | Risk assessment and awareness |  |
|  |  | Human-dog bond |  |
|  |  | Dog body language |  |
|  |  | General mental health |  |
|  |  | Condition/diagnosis-specific training |  |
|  |  | Safeguarding |  |
|  |  | Other (please specify) |  |
|  |  | No |  |
| **23** | **Are there any health checks the dogs need to have passed before getting involved in DAI in your organisation?**  **Yes No/Not Sure**  **[If yes] What are the health checks the dogs need to have passed before getting involved in DAI in your organisation?** | Annual vaccinations |  |
|  |  | Regular worming programme followed |  |
|  |  | Not receiving any veterinary prescribed medication |  |
|  |  | No current health issues |  |
|  |  | Regular grooming schedule followed |  |
|  |  | Not on a raw food diet |  |
|  |  | Other (please specify) |  |
|  |  | None |  |
| **24** | **Is the dog on a leash during a typical DAI session?** | Yes |  |
|  |  | No |  |
|  |  | Sometimes |  |
| **25** | **Is there a process for reporting inappropriate dog behaviour or injuries to human participants/the dog?** | Yes (optional to provide further information) |  |
|  |  | No |  |
|  |  | Not sure |  |
| **Dog characteristics** | | | |
| **26** | **Using the Kennel Club groups, what type of dogs are typically involved in your DAIs?**  ***Please follow this link for more information (if required):**<https://www.thekennelclub.org.uk/breed-standards/>  Please select **all** that apply **or** 'Not sure' if you don't know the answer to this question. | Hound group |  |
|  |  | Working group |  |
|  |  | Terrier group |  |
|  |  | Gundog group |  |
|  |  | Pastoral group |  |
|  |  | Utility group |  |
|  |  | Toy group |  |
|  |  | State other specific breed (optional) |  |
|  |  | Cross breeds |  |
|  |  | Not sure |  |
| **27** | **What is the youngest age a dog will start DAIs? (not including training)** | Rolling 2 months - 24 months |  |
|  |  | > 2 years |  |
|  |  | Not sure |  |
| **28** | **What is the oldest age a dog will normally work until?** | Rolling from 2 years |  |
|  |  | No criteria |  |
|  |  | Not sure |  |
| **About the Dog** | | | |
| **The following questions are related to dog welfare and wellbeing within your organisation.** | | | |
| **29** | **How many handlers, on average, do the dogs have whilst being involved in a DAI organisation?** | Rolling (1-10) |  |
|  |  | Other (please specify) |  |
| **30** | **On average, how frequently is a dog involved in a DAI?** | Daily |  |
|  |  | A few days a week |  |
|  |  | Weekly |  |
|  |  | Less than once a week |  |
| **31** | **Does your organisation stipulate a maximum number of hours a dog can participate in a DAI per week?** | Yes (please specify) |  |
|  |  | No |  |
|  |  | Not sure |  |
| **32** | **What strategies are in place to ensure the dog’s wellbeing is monitored during and after a DAI session? Please select all that apply or ‘the dog’s wellbeing is not monitored’.** | Handler observation |  |
|  |  | Veterinary observation  (specify how often) |  |
|  |  | Using checklists or other written materials |  |
|  |  | Access to water |  |
|  |  | Access to a place the dog can choose to go by themselves |  |
|  |  | Other (please specify) |  |
|  |  | The dog’s wellbeing is not monitored |  |
| **33** | **How long is a single DAI session on average?** | 30 minutes or less |  |
|  |  | 31 minutes – 1 hour |  |
|  |  | Over 1 hour, but less than 2 |  |
|  |  | 2 hours or more |  |
|  |  | Not sure |  |
| **Dog selection and training** | | | |
| **The following questions ask about the selection and training of dogs involved in your organisation.** | | | |
| **34** | **What training does a dog receive before starting DAIs? Please select all that apply.** | Obedience training (e.g., leadwork, sit, down etc) |  |
|  |  | Handling training (e.g., to be stroked/touched by strangers) |  |
|  |  | Greeting training (to greet people calmly) |  |
|  |  | Human-dog bond |  |
|  |  | Trick training |  |
|  |  | Socialisation training in environments likely to work in |  |
|  |  | Other (please specify) |  |
| **35** | **How is a dog selected for DAI work in your organisation? Please select all that apply.** | Temperament/behaviour assessment |  |
|  |  | Pass obedience test (e.g., Kennel Club test) |  |
|  |  | Health status (pass vet assessment) |  |
|  |  | Specific characteristics (e.g., size, colour, breed, age) (please specify) |  |
|  |  | Other (Please specify) |  |
| **Thank you very much for your time. Your information has made a valuable and much appreciated contribution to this research.** | | | |
